# Anti-Influenza Virus Activity and Constituents Characterization of *Paeonia delavayi* Extracts

**DOI:** 10.3390/molecules21091133

**Published:** 2016-08-26

**Authors:** Jinhua Li, Xianying Yang, Linfang Huang

**Affiliations:** Institute of Medicinal Plant Development, Peking Union Medical College & Chinese Academy of Medical Sciences, Beijing 100193, China; JinhuaLi108@126.com (J.L.); xyyangyn@126.com (X.Y.)

**Keywords:** *Paeonia delavayi*, neuraminidase inhibition, chemical constituents, anti-influenza activity, UPLC-Q-TOF-MS

## Abstract

*Paeonia delavayi,* an endemic species in southwestern China, has been widely used as a traditional remedy for cardiovascular, extravasated blood, stagnated blood and female diseases in traditional Chinese medicine (TCM). However, there are no reports on the anti-influenza virus activity of this species. Here, the anti-influenza virus activity of *P. delavayi* root extracts was first evaluated by an influenza virus neuraminidase (NA) inhibition assay. Meantime, constituents in the active extracts were identified using ultra-high performance liquid coupled with quadrupole time-of-flight mass spectrometry (UPLC-Q-TOF-MS) and seven major identified constituents were used to further evaluate the NA inhibitory activity. The results showed that the ethyl acetate fraction (EA) and the ethanol fraction (E) of *P. delavayi* both presented strong NA inhibitory activity with IC_50_ values of 75.932 μg/mL and 83.550 μg/mL, respectively. Twenty-seven constituents were characterized in these two active extracts by UPLC-Q-TOF-MS analysis, and seven major identified constituents exhibited high activity against the influenza virus. Among them, Benzoylpaeoniflorin (IC_50_ = 143.701 µM) and pentagalloylglucose (IC_50_ = 62.671 µM) exhibited the highest activity against the influenza virus, even far stronger than oseltamivir acid (IC_50_ = 281.308 µM). This study indicated that *P. delavayi* was a strong NA inhibitor, but cell-based inhibition, anti-influenza virus activity in vivo and anti-influenza virus mechanism still need to be tested and explored.

## 1. Introduction

The Paeonia genus, known for its ornamental value and medicinal properties, has a long medicinal history in Traditional Chinese Medicine (TCM) [1]. Pharmacological studies of this genus have shown a variety of activities, including analgesic, sedative, anti-inflammatory, antimicrobial, antitumor, antiviral, cardiovascular protection activities and so on [2,3,4]. The pharmacological activities are primarily attributed to monoterpenes, which has a “cage-like” pinane skeleton. Paeoniflorin and its derivatives represent major monoterpene glycosides in the plants of Paeonia. Furthermore, previous chemical investigations on this genus have led to the identification of seven classes, including monoterpenoid glycosides, flavonoids, tannins, stilbenes, triterpenoids and steroids, paeonols, and phenols [3,4]. *Paeonia delavayi*—one of the most important species in the Paeonia genus—is protected as an endemic species in southwestern China, which is mainly distributed in Yunnan, Sichuan and Tibet areas [5,6]. Its root, one of the main sources of the Chinese medicine “mudanpi”, has been used as a remedy for cardiovascular, extravasated blood, stagnated blood and female diseases [7,8].

The influenza virus, which belongs to the Orthomyxoviridae family, is a respiratory pathogen that reproduces rapidly, mutates frequently, and occasionally crosses the species barrier with high morbidity and mortality [9,10,11]. So far, two classes of drugs are available for influenza infections: M2 protein blockers (adamantanes), neuraminidase (NA) and inhibitors (zanamivir and oseltamivir) [12]. The M2 protein blockers, amantadine and rimantadine, are exclusively active against the influenza A virus, and possess adverse drug resistance effects. As a result, this class of drugs was strongly resisted by the Centers for Disease Control and Prevention (CDC) [13]. NA inhibitors are effective on both influenza A and B infections, and recommended by the WHO [14]. Nevertheless, the use of this class of antiviral drug has its limitations, such as drug-resistance, toxicity and cost [15,16,17,18]. Hence, new antiviral agents with high efficiency and low toxicity against the influenza virus are urgently needed.

Plants have been used for treating infectious diseases caused by viruses in traditional Chinese medicine for a long time. Much research has shown that plant extracts and natural products have a wide range of pharmacological activities, including anti-inflammatory, antibacterial, anticancer and antiviral [12]. For instance, polyphenols from green tea have the ability to inhibit influenza virus replication [19,20] and polyphenolic compounds derived from *Phellinus baumii* could inhibit influenza virus infection [10]. In recent years, the antiviral activity of some species of Paeonia has been explored. It has been reported that the extract of *P. lactiflora* possesses anti-influenza activity and has the potential for development as an anti-influenza agent [2]. In addition, literature also reported that *P. lactiflora* and *P. suffruticosa* both showed antiviral activities against respiratory syncytial virus (RSV) [21]. However, there are no reports of anti-influenza virus activity in *P. delavayi*. In the present study, an influenza virus NA inhibition assay was used to screen the anti-influenza virus activity of the extracts and UPLC-Q-TOF-MS was used to identify chemical constituents of the active extracts. Then, the anti-influenza virus activity of seven major constituents of extracts was further verified by determining their NA inhibitory capacity.

## 2. Results and Discussion

### 2.1. UPLC-Q-TOF-MS Analysis

**Figure 1:**
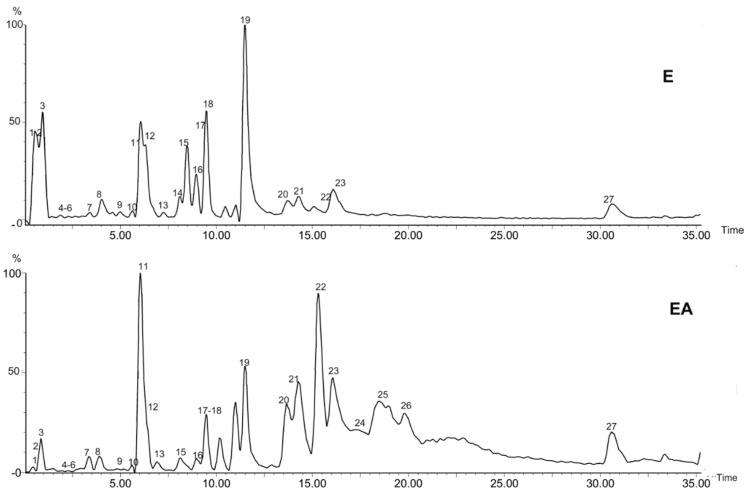
Base peak chromatogram (BPC) of the ethanol fraction (E) and the ethyl acetate fraction (EA) of *P.* *delavayi* root (negative mode).

**Figure 2:**
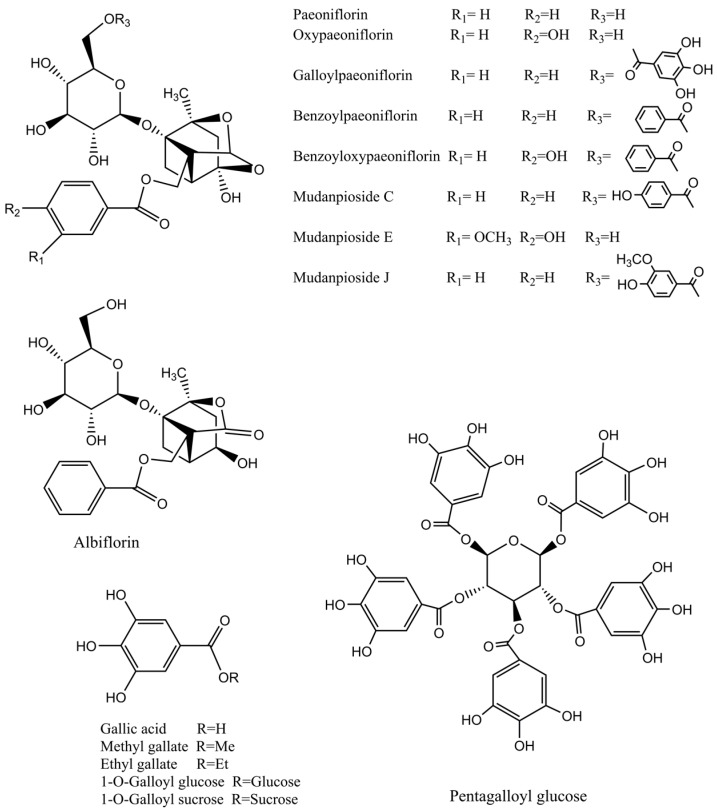
Chemical structures of the main identified constituents in the extracts from *P. delavayi* root.

According to the retention time and the molecular ions, together with the major fragments observed in MS spectra and followed by searching the literature, 27 compounds were tentatively identified. The identified compounds were definitely classified into three groups: phenols, tannins and monoterpene glycosides. The retention time, molecular formula, exact mass, experimental mass, the major fragments and identified compounds were listed in Table 1. The base peak ion (BPI) chromatogram and the chemical structures of the identified compounds were presented in Figure 1 and Figure 2, respectively.

Based on accurate molecular ions, fragment ions and the literature, three phenols and fourteen tannins were characterized in the extracts, all of which belong to gallic acid derivatives showing similar fragments, such as *m/z* at 169[gallic acid–H]^−^, 125[gallic acid–H–CO_2_]^−^. The ion [M–H]^−^ at *m/z* 183, 197 combined with neutral losses of 15 Da (CH_3_) and 29 Da (CH_2_CH_3_), respectively. The fragment ions of gallic acid, methy gallte and ethy gallte are consistent with previous reports [1,25]. Moreover, twelve galloylglucoses containing a glucose and different numbers of gallic acid moieties were detected, including the isomers of trigalloyl glucose, tetragalloyl glucose and hexagalloyl glucose. These results revealed that tannins were rich in the extracts.

Monoterpene glycosides are the major bioactive constituents of Paeonia species that have been reported by numerous literatures [4,26,27]. In this work, ten monoterpene glycosides were characterized, including albiflorin, paeoniflorin, oxypaeoniflorin, 4-O-ethylpaeoniflorin, benzoyloxypaeoniflorin, benzoylpaeoniflorin, galloylpaeoniflorin, mudanpioside C, mudanpioside E and mudanpioside J. These monoterpene glycosides have a pinane skeleton and a mono-glucose moiety and their fragmentation pattern is consistent with previous reports [22,23].

### 2.2. Influenza Virus Neuraminidase (NA) Activity Assay

**Figure 3:**
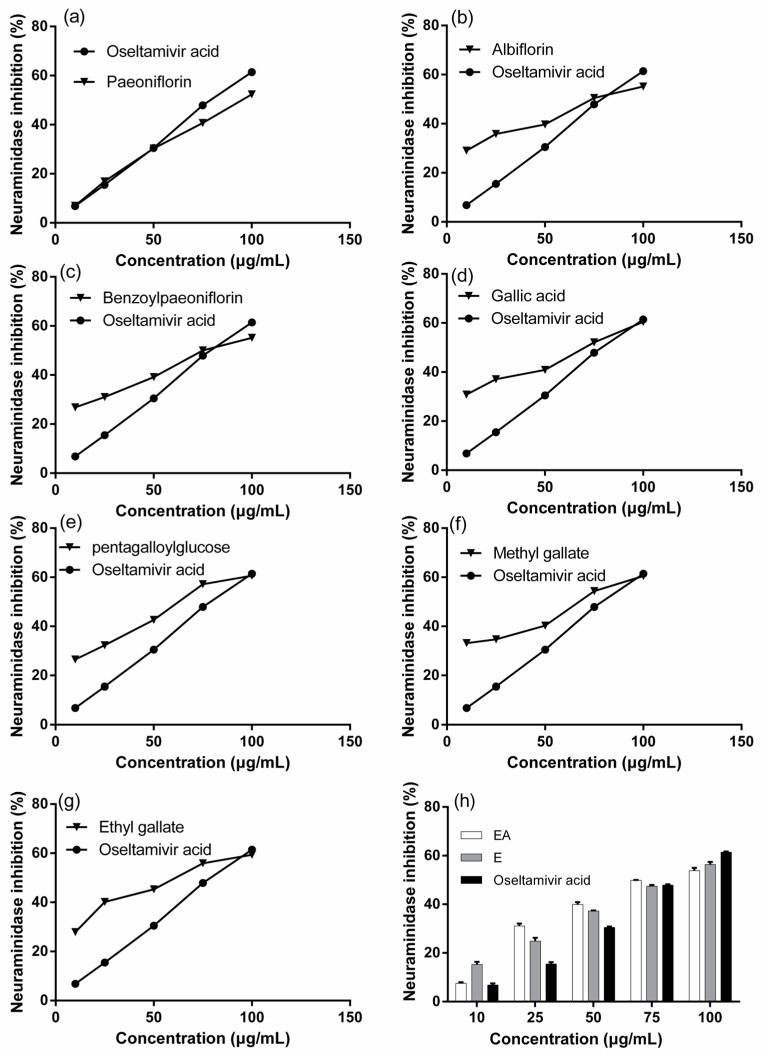
NA inhibitory activity of seven major constituents and the extracts of *P. delavayi* root. EA, the ethyl acetate fraction; E, the ethanol fraction.

NA has been regarded as one of the most important targets to screen the drugs of anti-influenza viruses A and B. The anti-influenza virus activity of the extracts and seven standard compounds were presented in Figure 3. They all showed dose-dependent activity and their IC_50_ values were shown in Table 2, and a lower IC_50_ value indicates a higher activity. Based on IC_50_ value, the activity of extracts was in the order of EA > Oseltamivir acid > E, and the activity of seven compounds was in the order of pentagalloylglucose > benzoylpaeoniflorin > albiflorin > paeoniflorin > oseltamivir acid > ethyl gallate > methyl gallat > gallic acid.

Although *P. delavayi* is one of the main sources of the Chinese traditional medicine “mudanpi”, which treats cardiovascular, extravasated blood, stagnated blood and female diseases; no research has reported its anti-viral activity. In the present study, the inhibitory activity of two extracts from *P. delavayi* (E and EA fractions) to NA was explored. Our findings, for the first time, showed that the extracts of *P. delavayi* display anti-influenza virus activity in vitro based on enzyme-based assay, especially EA fractions. Therefore, this study implies that *P. delavayi* may be used as a promising anti-viral drug resource, but its anti-influenza virus activity still needs to be further evaluated.

In order to confirm the bioactive constituents of the extracts, seven standard compounds that were identified in the qualitative assay (gallic acid, methyl gallate, ethyl gallate, pentagalloylglucose, benzoylpaeoniflorin, albiflorin, paeoniflorin) were used to analyse NA inhibitory capacity in vitro. These compounds belong to phenols, tannins and monoterpene glycosides (shown in Table 2). Oseltamivir acid, a metabolite of Oseltamivir, was used as a positive control. The result of the enzyme inhibition assay showed that all seven standard compounds exhibited anti-influenza virus activity, and pentagalloylglucose, benzoylpaeoniflorin, albiflorin and paeoniflorin showed stronger activity against NA than Oseltamivir acid. In several literature reports [28,29,30], the antiviral activities of pentagalloylglucose and gallic acid have been reported, but there are few reports regarding the antiviral activities of methyl gallate, ethyl gallate, benzoylpaeoniflorin, albiflorin and paeoniflorin. In this study, the NA inhibitory activity of methyl gallate, ethyl gallate, benzoylpaeoniflorin, albiflorin and paeoniflorin were reported for the first time.

Influenza is a highly contagious disease and can cause high morbidity and mortality in an epidemic. Antiviral agents are an important part of a rational approach to epidemic influenza and are critical to prevent pandemic influenza, but the resistance of influenza to antiviral drugs is now widespread due to mutations in the influenza viruses [15]. Hence, development of new natural anti-influenza drugs becomes necessary and urgent. Data presented here shows that extracts from *P. delavayi* are potent NA inhibitors. However, false-positive results in the commonly used method of enzyme-based NA inhibition assays were reported in several literatures and the reliability of a large number of flavonoid-based NA inhibitors reported in the literature is under question [31,32]. So, cell-based assay and anti-influenza virus activity, in vivo of the extracts and major components, need to be further tested and evaluated. Furthermore, high-throughput biology has greatly contributed to natural product-based drug discovery and some natural products were shown to be able to lead to structures for drug development, but these were false positives in most cases and those compounds acted as pan-assay interference compounds (PAINS) [33,34]. Gallic acid and some other phenolic natural products have been characterized promiscuous inhibitors because their target-based activities were linked to cell-based activities and activities in vivo, but there may be no common mechanisms involved [34]. Therefore, the study of the anti-influenza virus mechanism of *P. delavayi* root becomes quite necessary.

## 3. Experimental Section

### 3.1. Chemicals and Standard Substances

Analytical grade reagents that were used for extraction were obtained from Beijing Chemical Plant Co. Ltd. (Beijing, China). LC-MS grade acetonitrile was purchased from Fisher Scientific (Beijing, China). De-ionized water was purified using a Milli-Q system (Millipore, Bedford, MA, USA).

Gallic acid, methyl gallate, ethyl gallate, pentagalloylglucose, paeoniflorin, albiflorin and benzoylpaeoniflorin were purchased from Chengdu Must Biotechnology Co. Ltd. (Sichuan, China). Oseltamivir acid was purchased from Medchem Express, LLC (Monmouth Junction, NJ, USA).

### 3.2. Neuraminidase Inhibitors Screen Kit

Neuraminidase Inhibitors Screen Kit (NO. P0309) was purchased from Beyotime Institute of Biotechnology Co. Ltd. (Shanghai, China), which contains 10 mL buffer, 1 mL NA, 1 mL fluorescent substrate and 1.2 mL Milli-Q water.

### 3.3. Plant Material Collection and Sample Preparation

Samples of *P.* *delavayi* were collected in Dali, located in central Yunnan, China, in September 2010. The samples were identified by one of the authors, Professor Linfang Huang, and the voucher specimens (NO. P201009-188) were deposited in the Herbarium of the Chinese Academy of Medical Science & Peaking Union Medicinal College.

The samples were powdered to a fine powder in a grinder. A total of 500 g of powder was extracted by infusion with 2.5 L of petroleum ether for 24 h. Then, the residue was extracted with 80% ethanol using reflux extraction 3 times, 2 h each time. The filtered extracted solutions were concentrated using a rotary evaporator to yield a crude extract. The crude extract was dissolved in ethanol and successively extracted with ethyl acetate using the method of liquid-liquid extraction, and the solvent was removed to obtain a dry form of the ethyl acetate fraction (EA) and ethanol fraction (E) using a rotary evaporator. These two fractions were used for assays because these fractions exhibited greater influenza NA inhibitory activity than other fractions in our preliminary experiments. These fractions were dissolved in methanol and filtered through 0.22 μm Nylon micropore membranes and used for UHPLC-Q-TOF-MS assays at concentrations (m/v) of 5 mg/mL.

### 3.4. UPLC-Q-TOF-MS Analysis

#### 3.4.1. Liquid Chromatography

UPLC analysis was performed using a Waters Acquity UPLC system, equipped with a binary solvent system, an automatic sample manger and a photodiode-array (PDA) detector. The column that was used for the chromatographic separation was an ACQUITY UPLC BEH C18 2.1 mm × 100 mm, 1.7 μm (Waters, Milford, MA, USA) at 30 °C. The conditions consisted of a gradient elution using aqueous formic acid 0.1% (v/v) as mobile phase A and acetonitrile as phase B at a flow rate of 0.3 mL/min. The following gradient was applied: 0–2 min, 5% B; 2–4 min, 5%–10% B; 4–10 min, 10%–15% B; 10–30 min, 15%–20% B and 30–35 min, 20%–30% B. The injection volume was 2 μL and the injection temperature was 15 ℃.

#### 3.4.2. Mass Spectrometry

Tandem mass spectrometry was performed with a hybrid quadrupole orthogonal time-of-flight mass spectrometer (Q-TOF-MS) (Waters, Milford, MA, USA) using an electrospray ionization source for the ionization of the target compounds. The operating parameters were as follows: capillary voltage of 3.0 kV (ESI^+^) or 2.2 kV (ESI^−^); sample cone voltage 35 kV; extraction cone voltage 4 kV, ion source temperature 100 °C, desolvation temperature 450 °C and desolvation gas (N2) flow of 800 L/h, and scan range, *m/z* 100–1200. The mass spectrometer was calibrated with sodium formate. Leucine-enkephalin was used as an external reference at a constant flow of 5 µL/min. The data was processed with Masslynx V4.1 software (Waters, Milford, MA, USA).

### 3.5. Neuraminidase (NA) Inhibition Assay

The NA inhibition assay was carried out in a 96-well microplate reader using a procedure given by kit instruction [35]. Reaction mixture containing 70 μL of reaction buffer solution, 10 μL of NA and 10 μL of standard samples of the extracts in 10% DMSO were added to each well. Vibration mixing was carried out for about 1 min and incubation at a temperature of 37 °C for 2 min so that the NA and standard samples of the extracts can be fully interacted. After which, 10 μL of fluorescent substrate was added to give a total of 100 μL reaction mixture. The entire mixture was thoroughly mixed by vibration for about 1 min and the plate was incubated at 37 °C for 20 min. The fluorescence was read on a microplate spectrophotometer (Molecular Device, Gemini EM, USA) with an excitation wavelength at 322 nm and an emission wavelength at 450 nm. Oseltamivoir acid was used as a positive control. The inhibition (%) was calculated using the formula:

NA Inhibitory activity (%) = [1 − (F_s_ − F_0_)/(F_m_ − F_0_)] × 100%

Fs was fluorescence intensity in the presence of the sample, F_0_ was the absorbance in the presence of the sample background, and Fm was the absorbance of the negative control (without the sample). The 50% inhibitory concentration (IC_50_) was determined by probit regression in SPSS.

### 3.6. Statistical Analysis

NA inhibition experiments were performed in triplicate. SPSS 19 (BM SPSS, Chicago, IL, USA) and Graph Pad Prism 6 (GraphPad, San Diego, CA, USA) were used for the statistical analysis of the data.

## 4. Conclusions

In summary, the anti-influenza virus effect of *P. delavayi* root extracts was first evaluated by an influenza virus neuraminidase (NA) inhibition assay and chemical constituents of the active extract were characterized by UPLC-Q-TOF-MS in this study. As a result, the extracts showed strong NA inhibition and twenty-seven compounds were identified. In addition, seven major constituents in the extracts all showed favorable NA inhibitory activity. Among them, pentagalloylglucose, benzoylpaeoniflorin, albiflorin and paeoniflorin displayed stronger inhibitory activity than Oseltamivir acid. This study indicates that *P. delavayi* is a strong NA inhibitor, and provides scientific evidence and a new idea for anti-influenza virus drug discovery, but cell-based inhibition, anti-influenza virus activity in vivo, as well as anti-influenza virus mechanism, still need to be tested and explored.

## Figures and Tables

**Table 1:** Twenty-seven chemical constituents identified in the ethyl acetate fraction (EA) and the ethanol fraction (E) of *P.* *delavayi* root extracts using UPLC-Q-TOF-MS.

Peak	T_R_ (min)	Formula	*m/z* Calculated	*m/z* Experimental	Assigned Identity	Fragment Ions	Extracts	Reference
1	0.48	C_13_H_16_O_10_	331.0660	331.0826	1-O-Galloyl glucose	169[M–H–glucosyl]^−^, 125[M–H–glucosyl–CO_2_]^−^	E,EA	[1]
2	0.82	C_7_H_6_O_5_	169.0132	169.0360	Gallic acid *	125[M–H–CO_2_]^−^	E,EA	[1,22]
3	1.01	C_19_H_26_O_15_	493.3928	493.1234	1-O-Galloyl sucrose	457[M–H–2H_2_O]^−^, 331[M–2H–glucosyl]^−^, 169[M–H–2glucosyl]^−^, 125[M–H–2glucosyl–CO_2_]^−^	E,EA	[22,23]
4	3.37	C_8_H_8_O_5_	183.0288	183.0517	Methyl gallate *	169 [M–CH_3_]^−^, 168[M–H–CH_3_]^−^, 124[M–H–CH_3_–CO_2_]^−^, 125[M–CH_3_–CO_2_]^−^	E,EA	[1,22]
5	3.93	C_20_H_20_O_14_	483.0769	483.0833	Digalloyl-hexose	331[M–H–galloyl]^−^, 313[M–H–galloyl–H_2_O]^−^, 271, 169[gallic acid–H]^−^, 125[gallic acid–H–CO_2_]^−^	E,EA	[23]
6	4.38	C_27_H_24_O_18_	635.0879	635.0823	Trigalloyl-glucose	483[M–H–galloyl]^−^, 395[M–H–galloyl–2CO_2_]^−^, 169[gallic acid–H]^−^, 125[gallic acid–H–CO_2_]^−^	E,EA	[1]
7	4.65	C_24_H_29_O_13_	525.1595	525.1633	Mudanpioside E	363[M–H–glucosyl]^−^, 359[M–benzoic acid–OH–CO]^−^, 277, 121[benzoic acid–H]^−^	E,EA	[1]
8	4.97	C_27_H_24_O_18_	635.0879	635.0812	Trigalloyl-glucose isomer	483[M–H–galloyl]^−^, 465[M–H–gallic acid]^−^, 313[M–H–2galloyl–H_2_O]^−^, 169[gallic acid–H]^−^, 125[gallic acid–H–CO_2_]^−^	E,EA	[1]
9	5.57	C_23_H_28_O_11_	479.1548	479.1605	Albiflorin *	449[M–H–CH_2_O]^−^, 435[M–H–CO2]^−^ , 357[M–H–benzoic acid]^−^, 283[M–glucose–OH]^−^, 121[benzoic acid–H]^−^	E,EA	[1,23]
10	5.98	C_27_H_24_O_18_	635.0879	635.0818	Trigalloyl-glucose isomer	465[M–H–gallic acid]^−^, 448, 197[ethyl gallate–H]^−^, 169[M169[gallic acid–H]^−^, 125[gallic acid–H–CO_2_]^−^	E,EA	[1]
11	6.02	C_9_H_10_O_5_	197.0445	197.0670	Ethyl gallate *	169[M–CH_3_CH_2_]^−^, [M–CH_3_CH_2_–OH]^−^, 125[M–CH_3_CH_2_–CO_2_]^−^	E,EA	[1]
12	6.32	C_23_H_2_8O_11_	479.1548	479.1591	Paeoniflorin *	449[M–H–CH_2_O]^−^, 431[M–H–CH_2_O–H_2_O]^−^, 327[M–H–benzoic acid–CH_2_O]^−^, 309[M–H–benzoic acid–CH_2_O–H_2_O]^−^, 165[M–H–benzoic acid–CH_2_O–glucosyl]^−^, 121[benzoic acid–H]^−^	E,EA	[22,24]
13	6.86	C_23_H_28_O_12_	495.1497	495.1548	Oxypaeoniflorin	449[M–OH–CH_2_O]^−^, 465[M–H–CH_2_O]^−^, 327[M–(p-hydroxybenzoyl)–CO]^−^, 165[M–(p-hydroxybenzoyl)–CO–glucosyl]^−^, 137[p-hydroxybenzoyl]^−^, 121[benzoic acid–H]^−^	E,EA	[1,23]
14	8.21	C_41_H_30_O_26_	937.0947	937.0488	Dihydroxymethyl benzoyl tetragalloyl glucose	787[M–H–dihydroxymethylbenzoyl]^−^, 615[M–H–2galloyl–H_2_O]^−^, 477, 393, 183[ethyl gallate–H]^−^, 169[gallic acid–H]^−^, 125[gallic acid–H–CO_2_]^−^	E	[1]
15	8.39	C_30_H_32_O_18_	787.0989	787.0790	Tetragalloyl glucose	635[M–H–galloyl]^−^, 617[M–H–gallic acid]^−^, 477, 465[M–H–galloyl–gallic acid]^−^, 393, 301, 169[gallic acid–H]^−^, 125[gallic acid–H–CO_2_]^−^	E,EA	[1]
16	8.94	C_30_H_32_O_19_	787.0989	787.0802	Tetragalloyl glucose isomer	635[M–H–galloyl]^−^, 617[M–H–gallic acid]^−^, 477, 465[M–H–galloyl–gallic acid]^−^, 393 , 331 [M–H–3galloyl]^−^, 301, 169[gallic acid–H]^−^, 125[gallic acid–H–CO_2_]^−^	E,EA	[1]
17	9.45	C_30_H_32_O_15_	631.1658	631.1658	Galloylpaeoniflorin	449[M–H–galloyl–CH_2_O]^−^, 169[gallic acid–H]^−^, 125[gallic acid–H–CO_2_]^−^, 121[benzoic acid–H]^−^	E,EA	[22,23]
18	9.46	C_34_H_28_O_22_	787.0989	787.0787	Tetragalloyl glucose isomer	635[M–H–galloyl]^−^, 617[M–H–gallic acid]^−^, 477, 465[M–H–galloyl–gallic acid]^−^, 393 , 331[M–H–3galloyl]^−^ 301, 169[gallic acid–H]^−^, 125[gallic acid–H–CO_2_]^−^	E,EA	[1]
19	11.48	C_41_H_32_O_26_	939.1098	939.0750	Pentagalloyl glucose *	787[M–H–galloyl]^−^, 769[M–H–gallic acid]^−^, 635[M–H–2galloyl]^−^, 617[M–H–galloyl–gallic acid]^−^, 469[M–3H–2galloyl–glucosyl]^−^, 393, 169[gallic acid–H]^−^, 125[gallic acid–H–CO_2_]^−^	E,EA	[23]
20	13.63	C_48_H_34_O_30_	1091.1208	1091.0715	Hexagalloyl glucose	939[M–H–galloyl]^−^, 769[M–H–galloyl–gallic acid]^−^, 469[M–3H–3galloyl–glucosyl]^−^, 393, 169[gallic acid–H]^−^, 125[gallic acid–H–CO_2_]^−^	E,EA	[1]
21	14.25	C_48_H_34_O_31_	1091.1208	1091.0712	Hexagalloyl glucose isomer	769[M–H–galloyl–gallic acid]^−^, 469[M–3H–3galloyl–glucosyl]^−^, 393, 197[ethyl gallate–H]^−^, 169[gallic acid–H]^−^, 125[gallic acid–H–CO_2_]^−^	E,EA	[1]
22	16.12	C_48_H_34_O_33_	1091.1208	1091.0708	Hexagalloyl glucose isomer	769[M–H–galloyl–gallic acid]^−^, 469[M–3H–3galloyl–glucosyl]^−^, 393, 197[ethyl gallate–H]^−^, 169[gallic acid–H]^−^, 125[gallic acid–H–CO_2_]^−^	E,EA	[1]
23	16.98	C_30_H_32_O_13_	599.1765	599.1752	Mudanpioside C	477[M–benzoic acid]^−^, 257 , 137[p-hydroxybenzoyl]^−^, 121[benzoic acid–H]^−^	E,EA	[1]
24	17.42	C_31_H_34_O_14_	629.1870	629.1805	Mudanpioside J	599[M–H–CH_2_O]^−^, 257, 121[benzoic acid–H]^−^	EA	[1,22]
25	19.34	C_25_H_32_O_11_	507.1861	507.1892	4-O-Ethylpaeoniflorin	385[M–benzoic acid]^−^, 121[benzoic acid–H]^−^, 103[benzoic acid–H–H_2_O]^−^	EA	[4,7]
26	20.18	C_30_H_32_O_13_	599.1765	599.1741	Benzoyloxypaeoniflorin	522, 447[M–H–CH_2_O–benzoic acid]^−^, 137[p-hydroxybenzoyl]^−^, 121[benzoic acid–H]^−^	EA	[1,24]
27	30.63	C_30_H_31_O_12_	583.1816	583.1792	Benzoylpaeoniflorin *	553[M–H–CH_2_O]^−^, 431[M–H–benzoic acid–CH_2_O]^−^ 165[M–H–benzoic acid–benzoyl–CH_2_O–glucosyl]^−^, 121[benzoic acid–H]^−^	E,EA	[24]

* Identified with a reference.

**Table 2:** The IC_50_ values of *P. delavayi* root extracts (E and EA) and seven major constituents in neuraminidase (NA) inhibition assay.

Compounds	IC_50_	Compound Classified
Paeoniflorin	210.786 µM	Monoterpene glycosides
Albiflorin	167.115 µM	Monoterpene glycosides
Benzoylpaeoniflorin	143.701 µM	Monoterpene glycosides
Gallic acid	373.289 µM	Phenols
pentagalloylglucose	62.671 µM	Tannins
Methyl gallate	338.285 µM	Phenols
Ethyl gallate	274.195 µM	Phenols
the ethyl acetate fraction (EA)	75.932 µg/mL	
the ethanol fraction (E)	83.550 µg/mL	
Oseltamivir acid (Postive control)	281.308 µM (79.990 µg/mL)	
